# Quantitative Radionuclide Imaging Analysis of Enhanced Drug Delivery Induced by Photoimmunotherapy

**DOI:** 10.3390/ijms22158316

**Published:** 2021-08-02

**Authors:** Winn Aung, Atsushi B. Tsuji, Aya Sugyo, Masayuki Fujinaga, Ming-Rong Zhang, Tatsuya Higashi

**Affiliations:** 1 Department of Molecular Imaging and Theranostics, Institute for Quantum Medical Science, National Institutes for Quantum and Radiological Science and Technology (QST-iQMS), Inage, Chiba 263-8555, Japan; sugyo.aya@qst.go.jp (A.S.); higashi.tatsuya@qst.go.jp (T.H.); 2 Department of Advanced Nuclear Medicine Sciences, Institute for Quantum Medical Science, National Institutes for Quantum and Radiological Science and Technology (QST-iQMS), Inage, Chiba 263-8555, Japan; fujinaga.masayuki@qst.go.jp (M.F.); zhang.ming-rong@qst.go.jp (M.-R.Z.)

**Keywords:** photoimmunotherapy (PIT), super-enhanced permeability and retention (SUPR), radionuclide imaging, single-photon emission computed tomography (SPECT), positron emission tomography (PET), indocyanine green

## Abstract

Photoimmunotherapy (PIT) is an upcoming potential cancer treatment modality, the effect of which is improved in combination with chemotherapy. PIT causes a super-enhanced permeability and retention (SUPR) effect. Here, we quantitatively evaluated the SUPR effect using radiolabeled drugs of varying molecular weights (*^18^*F-5FU, *^111^*In-DTPA, *^99m^*Tc-HSA-D, and *^111^*In-IgG) to determine the appropriate drug size. PIT was conducted with an indocyanine green-labeled anti-HER2 antibody and an 808 nm laser irradiation. Mice were subcutaneously inoculated with HER2-positive cells in both hindlimbs. The tumor on one side was treated with PIT, and the contralateral side was not treated. The differences between tumor accumulations were evaluated using positron emission tomography or single-photon emission computed tomography. Imaging studies found increased tumor accumulation of agents after PIT. PIT-treated tumors showed significantly increased uptake of *^18^*F-5FU (*p* < 0.001) and *^99m^*Tc-HSA-D (*p* < 0.001). A tendency toward increased accumulation of *^111^*In-DTPA and *^111^*In-IgG was observed. These findings suggest that some low- and medium-molecular-weight agents are promising candidates for combined PIT, as are macromolecules; hence, administration after PIT could enhance their efficacy. Our findings encourage further preclinical and clinical studies to develop a combination therapy of PIT with conventional anticancer drugs.

## 1. Introduction

Cancer remains one of the most devastating diseases, with 18.1 million new cases and 9.6 million deaths worldwide in 2018 alone [1]. Currently, first-line cancer treatment regimens include one or more modalities, such as surgery, radiotherapy, immunotherapy, and chemotherapy. Therefore, imaging/therapeutic/theranostic agent delivery remains an important issue in cancer management. Generally, these agents can be passively distributed to target cancer cells in tumors, and passive targeting relies mostly on the enhanced permeability and retention (EPR) effect derived from the leaky nature of tumor vasculature [2]. The basic pathophysiological phenomenon of EPR is hyperpermeable tumor vasculature combined with impaired lymphatic drainage, resulting in enhanced permeability and retention of molecules [3,4]. Although the conventional EPR effect results in improved delivery to tumors compared with normal tissue, some tumors do not exhibit the EPR effect and the permeability of vessels may not be the same throughout a single tumor [5,6]. Moreover, the rapid proliferation of cancer cells around the vessels may impede therapeutic molecule penetration [7]. Therefore, the killing of perivascular cancer cells will help improve the EPR effect. Treatments that induce apoptosis or necrosis in perivascular tumor sheaths can enhance the delivery of therapeutic molecules to solid tumors [8]. Several approaches are being explored to enhance vascular permeability and augment drug delivery within tumors, such as radiotherapy [9,10], hyperthermia [11], and photoimmunotherapy (PIT) [12].

Recently, PIT using a photosensitizer-conjugated antibody against tumor-associated antigens accompanied with exposure to light of a specific wavelength has been attracting attention as a new type of therapeutic modality for cancer [12,13]. The photosensitizer–antibody conjugates strongly bind to cells in the immediate perivascular space, and thus PIT rapidly induces necrotic cell death, especially in the perivascular space, leading to a dramatic increase in tumor vascular permeability. This effect has been termed the “super-enhanced permeability and retention” (SUPR) effect [14]. Previous studies attempted to visualize and evaluate the SUPR effect using two imaging methods. Kobayashi et al. showed increased delivery of antibodies and non-targeted nanoparticles of large sizes through near-infrared fluorescence imaging [15,16] and magnetic resonance imaging (MRI) contrast agents [17] in PIT-treated tumors of mice. Compared with the near-infrared fluorescence imaging and MRI methods, radionuclide imaging is favorable in terms of its sensitivity and reliable quantification. However, a quantitative analysis of the SUPR effect using radionuclide imaging has not been performed.

**Figure 1:**
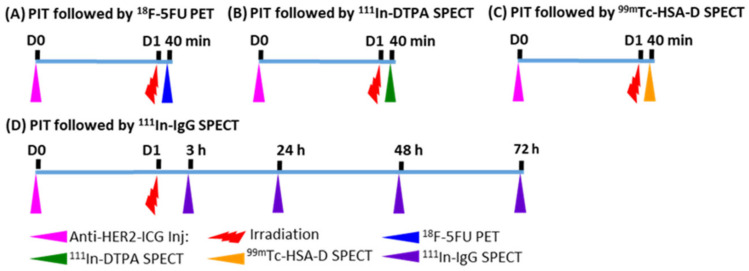
Experimental treatment scheme. (**A**) ^18^F-5FU PET imaging at 40 min after NIR-PIT; (**B**) ^111^In-DTPA SPECT imaging at 40 min after NIR-PIT; (**C**) ^99m^Tc-HSA-D SPECT imaging at 40 min after NIR-PIT; (**D**) ^111^In-IgG SPECT imaging at 3, 24, 48, and 72 h after NIR-PIT. Intravenous injection of anti-HER2-ICG into mice was performed one day before PIT.

Meanwhile, due to the limited therapeutic effect of PIT alone, the combination of anticancer agents with PIT is an option to improve the therapeutic outcome. Therefore, selection of drug molecule sizes that are suitable for enhanced permeability is also important for optimizing therapeutic efficacy. Thus, a methodology that supports investigation of drug delivery of varying molecular weights coupled with PIT is desirable. Here, we radiolabeled four molecules with different molecular weights and performed imaging and quantitative analysis of their accumulation in tumors after near-infrared PIT (NIR-PIT). Our radionuclide imaging study revealed that the SUPR effect could be observed for some low and medium molecules, as well as macromolecules, in tumors after PIT and suggested that these agents are promising candidates for combined PIT. The four non-targeted radioprobes used in this study were fluorine-18-fluorouracil (^18^F-5FU)—a positron emission tomography (PET) probe—and three single-photon emission computed tomography (SPECT) probes: indium-111-diethylenetriaminepentaacetic acid (^111^In-DTPA), technetium-99m-human serum albumin-diethylenetriamine pentaacetic acid (^99m^Tc-HSA-D), and indium-111-immunoglobulin G (^111^In-IgG)). Active drug delivery effect was excluded by using these non-targeted radioprobes. NIR-PIT was conducted with the fluorescent NIR-photosensitizer indocyanine green (ICG)-labeled anti-human epidermal growth factor receptor 2 (HER2) antibody and an 808-nm laser irradiation system. The schematic of the experiments is shown in Figure 1.

## 2. Results

### 2.1. Tumor Accumulation of HER2-ICG Visualized by NIR Fluorescence Imaging

**Figure 2:**
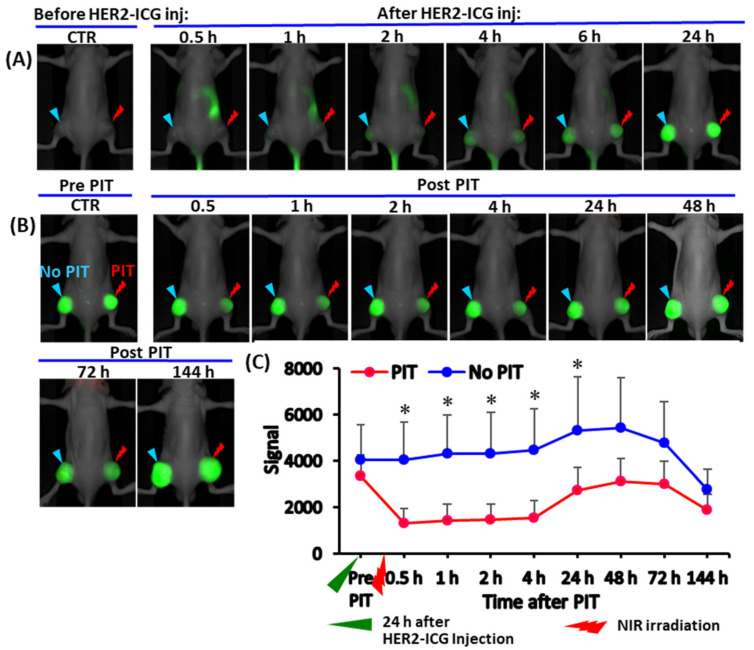
Serial whole-body NIR fluorescence images of a representative mouse bearing a xenografted tumor derived from an HER2-expressing A4 cell line. (**A**) Representative images at indicated time points (0.5, 1, 2, 4, 6 and 24 h) after intravenous injection of HER2-ICG (100 μg). (**B**) Images of the same mouse were obtained pre- and post-photoimmunotherapy (PIT) at 0.5, 1, 2, 4, 42, 48, 72 and 144 h. The right tumor received NIR irradiation (PIT) and the left tumor was an untreated control tumor (no PIT). (**C**) Line graph showing the fluorescence signal intensity (SI) of HER2-ICG in tumors from each side. SI in the treated tumors decreased initially after PIT and increased again after 24 h, suggesting photobleaching of dye and cancer cell death. Data are presented as means ± SD (*n* = 5), * *p* < 0.05 for PIT vs. no PIT. The green arrowhead indicates the time 24 h after the administration of HER2-ICG, and the red arrowhead indicates the day of NIR exposure.

The distribution and accumulation of HER2-ICG used for NIR-PIT of tumors was explored via fluorescence signal intensity (SI). After intravenous injection of HER2-ICG (100 μg), serial NIR fluorescence imaging showed that the HER2-ICG conjugate gradually and specifically accumulated in tumors, while it was washed out from the body. Antibody conjugate accumulation in tumors occurred within a few hours after injection, reached a maximum at 24 to 48 h and remained fairly constant until 144 h (Figure 2A–C). At 24 h after administration, a single dose of NIR light (50 J/cm^2^) was irradiated to the right tumor, while the left tumor was used for the un-irradiated control. The fluorescence SI of tumors that received PIT decreased immediately after receiving the irradiation, but it increased after 24 h (Figure 2B,C). The initial decrease in SI was probably caused by some degree of photobleaching of the probe and washout from necrotic cancer cells. A similar phenomenon was observed by Sano et al. [15].

### 2.2. PIT Effects on Tumor Growth in NUDE Mice

**Figure 3:**
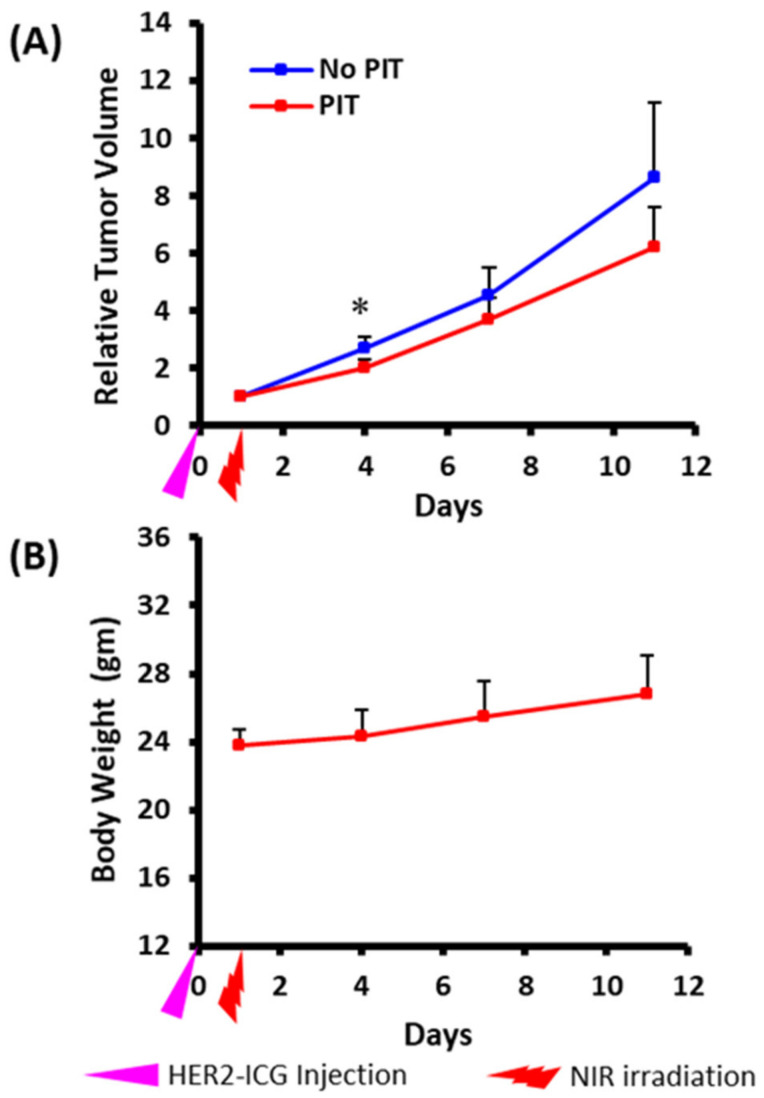
Photoimmunotherapeutic effects on tumor and body weight changes in mice. (**A**) Tumor volume change is shown as the ratio of the volume on the indicated day and the volume one day before the start of photoimmunotherapy (PIT). Tumors treated by NIR (PIT) (red) showed slow growth rates compared to contralateral tumors that received no treatment (no PIT) (blue). The difference was significant at day three after PIT. Values shown represent means ± SD (*n* = 5), * *p* < 0.05 for PIT vs. no PIT. (**B**) There was no average body weight loss. The pink arrowhead indicates the day of administration of HER2-ICG and the red arrowhead indicates the day of NIR exposure.

Tumor growth was inhibited by NIR-PIT and the chronological changes in relative tumor volume in PIT-treated tumors were smaller than those in control tumors. The difference in relative tumor volume was significant on day three after PIT treatment (Figure 3A). Body weight change (Figure 3B), skin damage and abnormal general conditions were not observed in these mice.

### 2.3. Increased 18F-5FU Uptake in PIT-Treated Tumors in PET/Computed Tomography (CT)

**Figure 4:**
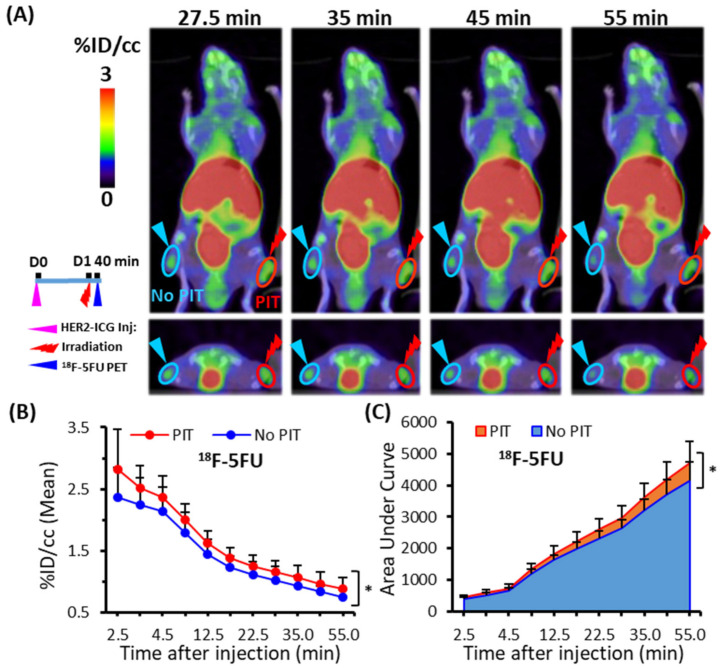
Dynamic PET/CT imaging of ^18^F-5FU in tumor-bearing mice. (**A**) Representative PET/CT image at four time points (27.5, 35, 45, and 55 min) after PIT. (**B**) Time-activity curve of average uptake of ^18^F-5FU. (**C**) Summed values of the areas under the curve (AUCs) for each time point were significantly increased in PIT-treated tumors (red) compared to untreated tumors (blue). Data are presented as means ± SD (*n* = 6), * *p* < 0.05 for PIT vs. no PIT.

Dynamic ^18^F-5FU PET imaging performed at 40 min after NIR light irradiation demonstrated significantly increased uptake (% injected dose per cc (% ID/cc)) of ^18^F-5FU in PIT-treated tumors compared to that in untreated tumors at all data-acquisition time points up to 55 min (Figure 4A,B). As a simplified measure of the radioactivity in tumors, the summed values of the *area under the time-activity curve* (AUC) covered up to each data-acquisition time point between 2.5 and 55 min were calculated and shown as % ID/cc * min. The AUCs of PIT-treated tumors were also significantly higher than those of untreated tumors (Figure 4C), and AUCs obtained at the representative data-acquisition time points are shown in Table 1.

### 2.4. Increased ^111^In-DTPA and ^99m^Tc-HSA-D Accumulation in PIT-Treated Tumors in SPECT/CT Imaging

**Figure 5:**
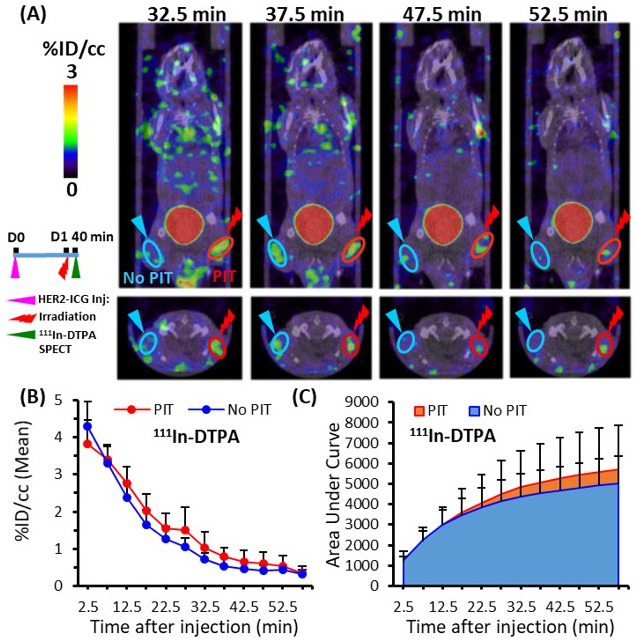
Dynamic SPECT/CT imaging of ^111^In-DTPA in tumor-bearing mice. (**A**) Representative SPECT/CT image at four time points (32.7, 37.5, 47.5, and 52.5 min) after PIT. (**B**) Time-activity curve of average accumulation of ^111^In-DTPA. (**C**) Summed values of the areas under the curve (AUCs) for each time point were evaluated. Uptake in the PIT-treated tumors (red) was compared with untreated tumors (blue). Data are presented as means ± SD (*n* = 7).

**Figure 6:**
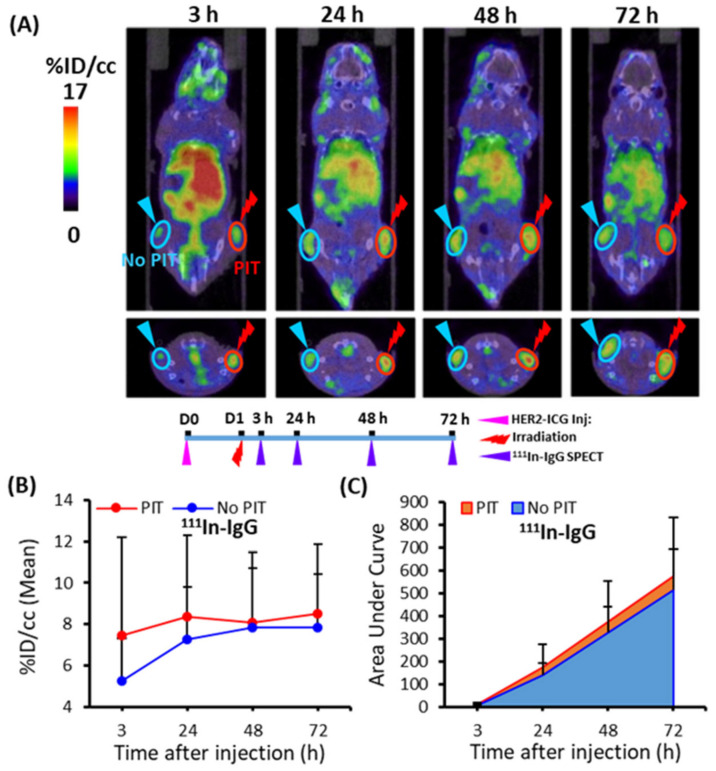
Dynamic SPECT/CT imaging of ^99m^Tc-HSA-D in tumor-bearing mice. (**A**) Representative SPECT/CT image at four time points (32.7, 37.5, 47.5, and 52.5 min) after PIT. (**B**) Time-activity curve of average accumulation of ^99m^Tc-HSA-D. (**C**) The summed values of the areas under the curve (AUCs) for each time point were significantly increased in PIT-treated tumors (red) compared to untreated tumors (blue). Data are presented as means ± SD (*n* = 8), * *p* < 0.05 for PIT vs. No PIT.

To assess the SUPR effect after PIT using a non-targeted SPECT imaging probe, the accumulation of the small molecule, ^111^In-DTPA, and the medium molecule, ^99m^Tc-HSA-D, in PIT-treated tumors was evaluated. Each probe was administered intravenously at 40 min after light irradiation, and an in vivo dynamic imaging study was performed. Only a tendency towards increased uptake and AUCs of ^111^In-DTPA was observed in the PIT-treated tumors in comparison to the control tumors (Figure 5A–C), but the significant differences were not noted. On the other hand, ^99m^Tc-HSA-D accumulation (Figure 6A,B) and the AUCs were significantly higher in PIT-treated tumors than in control tumors (Figure 6C). The AUCs for the representative data-acquisition time points are listed in Table 1.

### 2.5. ^111^In-IgG Accumulation in PIT-Treated Tumor in SPECT/CT Imaging

**Figure 7:**
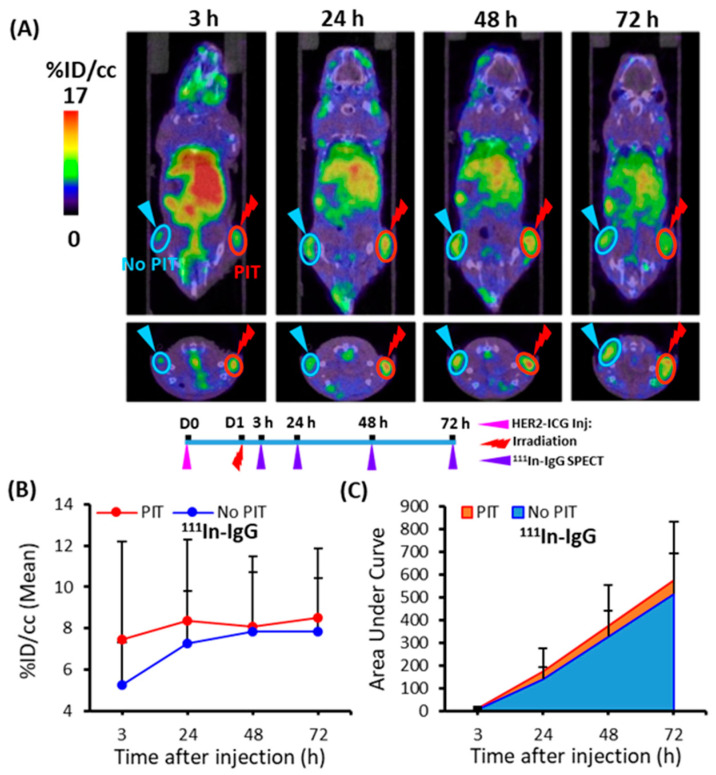
Temporal SPECT/CT imaging of ^111^In-IgG in tumor-bearing mice. (**A**) Representative SPECT/CT images at 3, 24, 48, and 72 h after intravenous injection of ^111^In-IgG are shown. The right tumor was irradiated with NIR light at 40 min before SPECT. (**B**) Time-activity curve of average accumulation of ^111^In-IgG. (**C**) Summed values of the areas under the curve (AUCs) for each time point were evaluated. These data showed a tendency for increased accumulation of ^111^In-IgG in PIT-treated tumors (red), although more sample numbers are required for the statistically significant difference. Data are presented as means ± SD (*n* = 8).

To demonstrate the PIT-induced SUPR effect for the non-specific large molecule probe ^111^In-IgG, tumor accumulation was examined for 3 days. At various time points (3, 24, 48, and 72 h), ^111^In-IgG accumulation and AUCs were comparatively higher in the PIT-treated tumors than in the untreated tumors (Figure 7). The tendency of increased accumulation of ^111^In-IgG in tumors subjected to PIT was noted, although a statistically significant difference was not observed (Table 1).

## 3. Discussion

The present study involved noninvasive radionuclide imaging to quantitatively assess the effect of SUPR induced by PIT. To visualize and quantify enhanced accumulation, non-specific radioprobes were used to exclude an active drug delivery effect. In this study, we conducted radionuclide imaging with four probes—^18^F-5FU, a low-molecular-weight (148 Da) PET probe; ^111^In-DTPA, a low-molecular-weight (504 Da) SPECT probe; ^99m^T- HSA-D, a medium-molecular-weight (66,492 Da) SPECT probe; and ^111^In-IgG, a high-molecular-weight (147,111 Da) SPECT probe—to quantify the SUPR effect induced by PIT. We set the suitable acquisition time for dynamic study as 60 min to assess the uptake of the radioprobes after the preliminary ^18^F-5FU imaging test. To decide the timing of the dynamic scan, we searched the relevant information and found that the SUPR effect was commonly seen from a few minutes to 1–2 h after irradiation using dynamic fluorescence imaging and MRI [15,16,17,18]. Based on these previous studies, we decided to conduct the radionuclide imaging at 40 min after irradiation. Concerning the **^111^**In-IgG imaging, the high background uptake of ^111^In-IgG at early time points led to a low tumor-to-background ratio and it was thus less suitable for imaging analysis. Therefore, we made the injection time identical to the other tracers and conducted the SPECT imaging as early as 3 h post-injection of ^111^In-IgG and at later time points.

The radionuclide imaging using the four probes showed a trend towards increased accumulation in the tumors treated with PIT compared to the untreated controls. Quantitative parameters, namely radioprobe accumulation and the AUCs of tumors, were significantly increased in ^18^F-5FU PET and ^99m^T-HSA-D SPECT after PIT (Figure 4 and Figure 6). A slight tendency for increased accumulation of ^111^In-DTPA and ^111^In-IgG in tumors was also noted, but there was no statistically significant difference. Based on our results, ^99m^Tc-HSA-D seems a good and convincing probe to use to trace the SUPR effect. The second-best probe was ^18^F-5FU. Moreover, a small increase of ^111^In-IgG and ^111^In-DTPA uptakes after PIT gave us some questions that need to be clarified in further studies. One probable reason for a small increase of ^111^In-IgG is conceivable: the SUPR effect continues for a short time, whereas IgG accumulation rate is very slow, and this mismatch could lead to the unexpected results obtained in the present study. Although the magnitude of the increase was small, the easy repeatable nature of PIT combined with repeated administration of drugs could be beneficial in some cancer management situations.

Our quantitative data provide evidence that PIT augments the permeability and retention of low- and medium-molecular-weight agents in cancer tissues. Most previous reports on the SUPR effect were conducted with high-molecular-weight drugs, as well as some with low- or medium-molecular-weight agents: tratuzumab-IRDye 800DX conjugate [15,19] and ICG [18] with dynamic fluorescence imaging; and Gd-labeled polyamidoamine dendrimer (G6-Gd) [15], Gd-chelated albumin macromolecules [17] and ultra-small paramagnetic iron oxide [15] with MRI. Although their accumulations increased after PIT, optical imaging and MRI are generally less appropriate for quantitative analysis. Therefore, the present study employed radionuclide imaging, which provides more reliable quantitative data. Our quantitative radionuclide imaging confirmed the advantages of small and medium agents for the SUPR effect, especially the albumin-based agent.

Concerning drug accumulation in tumor tissue, a balance between the molecular weight of the drugs and the gaps or fenestrations in capillaries is important: low-molecular-weight probes readily leak and high-molecular-weight probes slowly leak from the vasculature. This might be one rationale behind the statistical significance observed in low- and medium-molecular-weight radioprobes. The significantly increased tumor accumulation of small and medium molecules after PIT could be involved in the duration of the tumor vascular permeability changes induced by PIT. Sano et al. stated that the SUPR effect was most obvious in the early hours after PIT; thereafter, it gradually returned to baseline by 24 h because of tissue repair processes of necrotic perivascular tumor cells that can recover tumor interstitial pressure [15]. Therefore, the earlier time points after PIT would be a more preferential window to grasp the SUPR effect. This is different from the conventional EPR effect and should be considered in the optimization of the combined PIT.

There are several limitations to the present study. First, the mechanism of probe accumulation in the SUPR consequence depends not only on the molecular weight but also on other factors; however, we focused on the role of molecular weight in this study. Therefore, it is necessary to evaluate the SUPR effect for specific therapeutic agents, especially with active drug delivery agents, such as molecular-targeted peptides and antibodies. Second, subcutaneous tumor models in mice are well-established and widely used in many types of oncologic research, but the nature of the tumor microenvironment does not completely reflect all characteristics of cancerous tissues in humans. Radionuclide imaging can noninvasively quantify radioprobes accumulation in humans; therefore, further clinical studies are required to clarify the role of the SUPR effect and enhance the efficacy of PIT in the future. Third, our study did not include control groups receiving the laser irradiation only and ICG plus irradiation, though we previously performed laser exposure only on a subcutaneous pancreatic tumor model and confirmed that there was no therapeutic effect on this tumor model [13].

In summary, the PIT-induced SUPR effect enhanced permeability and took advantage of a more homogeneous and drastic distribution of therapeutic agents in tumors through which a better, combined therapeutic effect compared to PIT alone can be anticipated. The radionuclide imaging approach was elucidated for the PIT-mediated SUPR effect and could help in optimizing therapeutic measures by clarifying the feasibility of selecting a drug size and monitoring its distribution. Our quantitative imaging analysis revealed that the SUPR effect was induced in some low- and medium-molecular-weight molecules, in addition to high-molecular-weight drugs. Amelioration of therapeutic regimens, such as optimization of the timing of drug administration, fine-tuning of irradiation dose, and photosensitizers, could be achieved with our approach in further clinical studies.

## 4. Materials and Methods

### 4.1. Cell Culture

The immortalized mouse fibroblast cell line A4, established from mouse 3T3 cells and transfected with a human epidermal growth factor receptor 2 (HER2)-expression vector, was used for the experiments. A4 cells were maintained in RPMI 1640 medium (Wako, Osaka, Japan) supplemented with 10% fetal bovine serum (Nichirei Biosciences, Tokyo, Japan), 100 U/mL penicillin G sodium, and 100 mg/mL streptomycin sulfate (Invitrogen, Carlsbad, CA, USA) at 37 °C in a humidified atmosphere containing 5% CO_2_.

### 4.2. Subcutaneous Tumor Mouse Model

A single-cell suspension of 2 × 10^6^ A4 cells in 100 µL of RPMI medium was subcutaneously inoculated into both hindlimbs of five-week-old male BALB/cA Jcl-nu/nu nude mice (CLEA Japan Inc., Shizuoka, Japan) for the in vivo studies. All animal experiments were conducted in accordance with the guidelines for animal experimentation approved by the Animal Care and Use Committee of our institution, the National Institutes for Quantum and Radiological Science and Technology.

### 4.3. Synthesis of ICG-Anti-HER2 Antibody

The human monoclonal antibody anti-HER2 (trastuzumab) was purchased from Chugai Pharmaceutical Co., Ltd. (Tokyo, Japan) and dissolved in water. An NH2 ICG Labeling Kit from Dojindo Molecular Technologies, Inc. (Rockville, MD, USA) was used for labeling of the antibody with ICG according to the instructions. A component of this kit, NH_2_-reactive ICG, has a succinimidyl ester group and can easily form a covalent bond with the amino group of the antibody. In brief, NH_2_-reactive dyes were mixed with an anti-HER2 antibody (150 μg) solution on the membrane of a filtration tube and incubated at 37 °C for 10 min. Then, the buffer solution was added to the mixture and centrifugation was done. The conjugate (HER2-ICG) was recovered by pipetting with phosphate-buffered saline. The concentration of the conjugates was measured by Bradford protein assay using a Bio-Rad microplate reader (Bio-Rad Laboratories, Inc., Hercules, CA, USA). The absorbance of ICG was measured at 800 nm using a Synergy HT multi-mode microplate reader (BioTek Instruments, Inc., Winooski, VT, USA). The number of dyes/antibody ratios was calculated to be approximately 0.6.

### 4.4. In Vivo NIR Fluorescence Imaging

One week after inoculation, subcutaneous A4 tumors in mice reached approximately 10 mm at the longest diameter, and tumor-bearing mice received intravenous injection with HER2-ICG (100 μg) via the tail vein. To assess the biodistribution and clearance of the conjugate, the mice were anesthetized through 2.5% isoflurane inhalation and in vivo NIR fluorescence imaging was performed. Spectral fluorescence images at the dorsal position of mice were acquired using the Pearl Trilogy Imaging system (LI-COR Biosciences, Lincoln, NE, USA) with the 800 nm channel at pre-injection, followed by various post-injection time points (0.5, 1, 2, 4, 6, and 24 h). Image analysis was conducted using the Image Studio Software. After selection of the appropriate parameters and setting a suitable background reading, the region of interest was drawn to encompass the tumor and the fluorescence SI was measured.

### 4.5. In Vivo PIT of Tumor

For NIR-PIT and further experiments, tumor-bearing mice were randomly assigned. The mean tumor size at the time of PIT was about 276.3 ± 81.9 mm^3^. Mice were initially injected with a HER2-ICG (100 μg) conjugate intravenously. On day 1, the right tumor was irradiated with NIR light from an Infrared Diode Laser system (Laser Create Co., Tokyo, Japan) at a wavelength of 808 ± 3 nm and a power density of 50 J/cm^2^, as measured with a StarLite Laser Power meter (OPHIR Japan Ltd., Saitama, Japan). The surrounding areas of the tumor were covered from light using aluminum foil during irradiation. Furthermore, post-PIT serial NIR fluorescence imaging and image analysis were performed at various time points (0.5, 1, 2, 4, 6, 24, 48, 72, and 144 h after PIT).

To examine the tumor response to PIT, tumors of mice (*n* = 5) were measured two times a week throughout the experiment using calipers, and volumes were approximated using the formula: volume (mm^3^) = (length (mm)) × (width (mm))^2^ × 0.5. Relative tumor volume was determined as the volume on the indicated day divided by the starting volume on the day prior to treatment. The body weight of mice was also weighed twice a week; general health conditions and local skin conditions at the light-exposed areas of the mice were monitored daily.

### 4.6. Preparation of Radiotracers

#### 4.6.1. Fluorine-18-Fluorouracil (^18^F-5FU)

Fluorouracil (5FU) is a pyrimidine analogue used as an antineoplastic agent [20]. ^18^F-5FU was radiosynthesized as described here. All chemicals and reagents were purchased from commercial sources. Iodonium ylide derivatives were synthesized as precursors from 5-iodo-2,4-dimethoxypyrimidine with spiroadamantyl-1,3-dioxane-4,6-dione, according to a previously reported procedure [21]. Fluorine-18, as a [^18^F]fluoride ([^18^F]^−^), was produced by the ^18^O(p, n) ^18^F reaction on over 98 atom percentage H_2_^18^O (ROTEM Industries, Arava, Israel) using 18 MeV protons (14.2 MeV on target). The ^18^F-labeling synthesizer was set to an automated multi-purpose radiosynthesis system developed in our institute. [^18^F]F^−^ was trapped on a Sep-Pak Light QMA cartridge (Waters), eluted from the cartridge with a solution of tetraethylammonium bicarbonate (4.5 mg) in acetonitrile (CH_3_CN)/H_2_O (*v/v*, 7/3, 1.0 mL), and then transferred to a reaction vial. The aqueous [^18^F]F^−^ solution was dried at 120 °C for 30 min to remove water and CH_3_CN. A solution of the precursor (4 mg) in DMF (600 μL) was added to the reaction vial and heated at 130 °C for 10 min. A solution of trifluoroacetic acid (100 μL) and 2 mol/mL sodium bromide (300 μL) was added to the reaction mixture and heated at 130 °C for 10 min. After the reaction was complete, the reaction solvent was removed and water (1 mL) was added. The reaction mixture was purified using an InertSustain AQ-C18 column (10 mm i.d. × 250 mm) with 20 mM phosphate buffer at 3 mL/min. The radioactive fraction of ^18^F-5FU was directly passed through a Sep-Pak Alumina N light (Waters) to obtain the final product. The results of a typical batch were as follows: synthesis time from EOB: 101 ± 7 min (*n* = 10); radiochemical yield (decay-corrected based on [^18^F]F^−^): 26 ± 15% (*n* = 10); radiochemical purity: >99%; molar activity at EOS: 110 ± 50 GBq/mol (*n* = 10). The approximate molecular weight of ^18^F-5FU was determined to be 148 Da.

#### 4.6.2. Technetium-99m Human Serum Albumin-Diethylenetriamine Pentaacetic Acid (^99m^Tc-HSA-D)

^99m^Tc-HSA-D (Poolscinti**^®^** Injectable) is a commercially available radiopharmaceutical used as a diagnostic agent for hemodynamic and vascular lesions [22]. It was purchased from Nihon Medi-Physics Co., Ltd. (Tokyo, Japan). Its approximate molecular weight was 66,492 Da.

#### 4.6.3. Radiolabeling of Diethylenetriaminepentaacetic Acid (DTPA) with Indium-111 (^111^In)

DTPA is widely used as a metal chelator in the development of radiopharmaceuticals and contrast agents [23]. A total of 3.3 pmol DTPA (Sigma-Aldrich, St. Louis, MO, USA) was mixed with 7.4 MBq of indium-111 chloride (^111^InCl_3_; Nihon Medi-Physics, Tokyo, Japan) in 0.5 M acetate buffer (pH 6.0), and the mixture was incubated for 30 min at room temperature. The radiolabeled DTPA was eluted with 0.1 M acetate buffer (pH 6.0) using a Sephadex G-50 column (GE Healthcare, Little Chalfont, UK) for purification. The radiochemical purity was 100%, as determined by thin-layer chromatography (Merck Millipore, Billerica, MA, USA), and the specific activity of ^111^In-DTPA was 5.69. The approximate molecular weight of ^111^In-DTPA was 504 Da.

#### 4.6.4. Radiolabeling of Immunoglobulin G (IgG) with Indium-111 (^111^In)

IgG is the most common type of antibody found in blood circulation [24]. Mouse IgG (Sigma-Aldrich, St. Louis, MO, USA) was conjugated with *p*-SCN-Bn-CHX-A″-DTPA (Macrocyclics, Dallas, TX, USA), as previously described [25]. The *p*-SCN-Bn-CHX-A″-DTPA-conjugated antibody was purified using a Sephadex G-50 (GE Healthcare) column (700× *g* for 2 min). The conjugation ratio of chelate to antibody was estimated to be approximately 1.5, as determined by cellulose acetate electrophoresis. The chelate-conjugated antibody (0.5 nmol) was mixed with 1.48 MBq of ^111^InCl_3_ (Nihon Medi-Physics) in 0.5 M acetate buffer (pH 6.0) and the mixture was incubated for 30 min at room temperature. The radiolabeled antibody was separated from free In-111 using a Sephadex G-50 column (700× *g* for 2 min once). The labeling yields of ^111^In-IgG ranged from 71.4% to 86.5%, the radiochemical purity was 100%, and the specific activity was 14.1 to 17.1 kBq/μg. The approximate molecular weight of ^111^In-IgG was 147,111 Da.

### 4.7. ^18^F-5FU PET Imaging

^18^F-5FU PET scans were conducted 40 min after NIR-PIT. Tumor-bearing mice (*n* = 6) were intravenously injected with ^18^F-5FU (3.7 MBq). Dynamic PET data acquisition was conducted for 60 min using a small-animal PET system (Inveon, Siemens, Knoxville, TN, USA) under 1.5% isoflurane anesthesia. Images were reconstructed using the maximum a posteriori (MAP) method with attenuation correction using Inveon Acquisition Workplace software (Siemens). Using PMOD software (version 3.5; PMOD Technologies GmbH, Zürich, Switzerland), the average radioprobe uptake in the tumors was measured from the volume of interest (VOI), which was manually positioned based on the tumor contour in the different orthogonal planes with the largest diameter. Tracer uptake in the tumor was decay-corrected to injection time, normalized to weights, and expressed as the percentage of the injected dose per cc (% ID/cc). Time-activity curves of ^18^F-5FU were determined. Summed values of the AUCs (% ID/cc × time) covered up to each time point were calculated appropriately. To provide the anatomic landscape of PET images, at the end of PET scans, CT scans of the same mice were immediately performed after using the small-animal CT system (R_mCT2; Rigaku, Tokyo, Japan). The scan conditions included radiation parameters of 200 µA and 90 kV, an FOV of 60 mm, and an acquisition time of 34 s. CT images were collected, reconstructed, and observed using the I-View-R software (Rigaku). Dynamic PET and CT images of the mice were coregistered to confirm the anatomic location of tumors and fused images were obtained using PMOD software.

### 4.8. ^111^In-DTPA and ^99m^Tc-HSA-D SPECT/CT Imaging

SPECT imaging was conducted 40 min after the NIR-PIT. Tumor-bearing mice from each group (^111^In-DTPA, *n* = 7; ^99m^Tc-HSA-D, *n* = 8) were intravenously injected with ^111^In-DTPA (7.4 MBq) and ^99m^Tc-HSA-D (7.4 MBq), respectively. Dynamic SPECT data acquisition was conducted for 60 min, using a VECTor/CT SPECT/CT Pre-Clinical Imaging system with a multi-pinhole collimator (MILabs, Utrecht, the Netherlands) under 1.5% isoflurane anesthesia. SPECT images were reconstructed using a pixel-based ordered-subsets expectation-maximization algorithm with eight subsets and two iterations on a 0.8-mm voxel grid without attenuation correction. Computed tomography scans were acquired with an X-ray source set at 60 kVp and 615 μA after the SPECT scan, and images were reconstructed using a filtered backprojection algorithm for the cone beam. Merged images were obtained using PMOD software (version 3.5; PMOD Technologies, Zurich, Switzerland). The VOI was drawn over tumors and radioprobe uptake was quantified as the % ID/cc, and AUCs covered up to each time point were also calculated appropriately.

### 4.9. ^111^In-IgG SPECT/CT Imaging

Mice (*n* = 8) were injected with 1.85 MBq of ^111^In-IgG into the tail vein. The injected protein dose was adjusted to 50 μg/mouse by adding an intact antibody. At 3, 24, 48, and 72 h after the injection, the mice were anesthetized through isoflurane inhalation and imaged using a VECTor/CT Imaging system (MILabs). Forty minutes before static SPECT imaging, the right tumor was irradiated with NIR light. SPECT data were acquired for 30 min. Acquisition and reconstruction of SPECT and CT images, merging of both images, drawing of the VOI of the encompassed tumor, and quantification of the probe uptake of the tumor were conducted using methods similar to those mentioned above. Tumor uptake at each time point was plotted against time, from which the AUC was calculated using PMOD software.

### 4.10. Statistical Analysis

All results are expressed as means ± SD. The differences in results (radioprobe uptake, AUCs) between groups were evaluated using ANOVA (two-factor with replication; Excel, Microsoft, Redmond, WA, USA). The data shown in Figure 2 and Figure 3 (fluorescence signal intensity, relative tumor volume) were evaluated by two-tailed paired *t*-testing. Statistical significance was set at *p* < 0.05.

## 5. Conclusions

The SUPR effect induced by PIT was visualized and quantified by radionuclide imaging using four non-targeted specific radioprobes. This report shows that the SUPR effect can be observed for some small and medium molecules, as well as macromolecules, in tumors after PIT through noninvasive and quantitative imaging. These agents are promising candidates for combined PIT. Being able to observe whether the SUPR effect is present is desirable in cancer management because it helps in selecting appropriate drugs and regimens that can improve the therapeutic outcomes.

## Figures and Tables

**Table 1:** Summary of super-enhanced permeability and retention (SUPR) effects.

Radioprobe	MW	Parameter	No PIT	PIT	No: of Mice	ANOVA Test
	Dalton		Mean ± SD	Mean ± SD	(*n*)	*p* Value
^18^F-5FU	148	AUC0~55 m	4150.1 ± 599.4	4721.5 ± 653.7	6	0.001 *
		(% ID/cc * min)				
^111^In-DTPA	504	AUC0~52.5 m	4926.4 ± 1906.2	5594.5 ± 2150.6	7	0.018
		(% ID/cc * min)				
^99m^Tc-HSA-D	66,492	AUC0~52.5 m	9376.7 ± 2311.3	13,596.4 ± 2735.2	8	0.001 *
		(% ID/cc * min)				
^111^In-IgG	147,111	AUC0~72 h	515.0 ± 177.7	574.1 ± 258.88	8	0.30
		(% ID/cc * h)				

Each radioprobe accumulation in PIT-treated tumors and untreated tumors was compared. * *p* < 0.05 = statistically significant. AUC0~55 m is the area under the time-activity curve between 0 and 55 min post-injection. AUC0~52.5 m is the area under the time-activity curve between 0 and 52.5 min post-injection. AUC0~72 h is the area under the time-activity curve between 0 and 72 h post-injection.

## Data Availability

All data generated or analyzed in this study are available from the corresponding author on reasonable request.

## Abbreviations

AUC	Area under the time-activity curve
CT	Computed tomography
DTPA	Diethylenetriaminepentaacetic acid
EPR	Enhanced permeability and retention
FBS	Fetal bovine serum
^18^F-5FU	Fluorine-18-fluorouracil
G6-Gd	Gd-labeled polyamidoamine dendrimer
HER2	Human epidermal growth factor receptor 2
HER2-ICG	Indocyanine green-conjugated anti-HER2 antibody
^111^InCl_3_	Indium-111 chloride
^111^In-DTPA	Indium-111-diethylenetriaminepentaacetic acid
IgG	Immunoglobulin G
^111^In-IgG	Indium-111-immunoglobulin G
ID/cc	Injected radioactivity dose per cubic centimeter
MAP	Maximum a posteriori
MRI	Magnetic resonance imaging
NIR-PIT	Near-infrared photoimmunotherapy
PBS	Phosphate-buffered saline
PET	Positron emission tomography
ROI	Region of interest
SI	Signal intensity
SPECT	Single-photon emission computed tomography
SUPR	Super-enhanced permeability and retention
^99m^Tc-HSA-D	Technetium-99m human serum albumin-diethylenetriamine pentaacetic acid
VOI	Volume of interest
